# From motivation to acceptability: a survey of public attitudes towards organ donation in Denmark

**DOI:** 10.1186/s13737-016-0035-2

**Published:** 2016-05-23

**Authors:** Francisca Nordfalk, Maria Olejaz, Anja M. B. Jensen, Lea Larsen Skovgaard, Klaus Hoeyer

**Affiliations:** Department of Public Health, Section for Health Services Research, University of Copenhagen, Øster Farimagsgade 5, Copenhagen K, DK 1014 Denmark

**Keywords:** Acceptability, Denmark, Organ donation, Public attitudes, Survey

## Abstract

**Background:**

Over the past three decades, public attitudes to organ donation have been a subject of numerous studies focusing on donor motivation. Here, we present a fresh approach. We suggest focusing on public acceptability instead of motivation. The point is to understand public attitudes well enough to avoid risking public support for organ transplantation. We conducted the study in Denmark because there have been significant developments in public attitudes to organ donation in this country. In the 1990s, Denmark was a country with very low public support for organ donation and Denmark was the last country in Europe to introduce brain death as a legal criterion of death, whereas today Eurobarometer surveys rate Denmark as one of the European countries with the highest support for deceased organ donation from brain dead donors.

**Methods:**

We conducted a telephone survey in Denmark (*N* = 1195). A questionnaire was developed on the basis of preceding qualitative studies and pilot testing and included reuse of one item from earlier surveys to facilitate historical comparison. The analysis of the data was carried out using IBM SPSS Statistics 22 and focused on descriptive statistics.

**Results:**

A clear majority of 91.9 % are positive or very positive towards organ donation; 85.8 % like the idea of their body being used after their death, 85.0 % is willing to donate their own organs, 82.1 % to donate their tissue and only 2.3 % find that too much has been done to promote organ donation. There is limited support for monetary incentives for organ donation (5.8 %) and presumed consent (30.4 %), while a majority (63.9 %) supports making it mandatory to register a personal decision. Religious self-identification has limited impact on attitudes.

**Conclusions:**

We can identify a shift over the past three decades from marked opposition to organ transplantation to strong support as well as a pattern in the contemporary public attitudes, which can help explain what is central to public acceptability: self-determination. Policies fostering choice are met with a majority of positive attitudes, while presumed consent and monetary incentives are met with more negative attitudes. Our approach calls for comparative studies in other countries to generate a better overall understanding of the conditions of acceptability, which need to be in place to ensure the long-term social robustness of organ donation and thereby safeguard this important medical technology.

## Background

Over the past three decades, public attitudes to organ donation have been a subject of numerous studies [1, 2]. In most cases, the interest in public attitudes is stimulated by the long waiting lists for organ transplants, and as a result, most studies focus on identifying the factors that may increase the willingness to donate [3] or aid the removal of presumed barriers [1, 4]. Often, studies build on the assumption that knowledge determines attitude, which in turn influences behaviour [5, 6]. Such studies face the problem of the oft-identified gap between attitude and behaviour, i.e. not everyone who is hypothetically in favour of organ donation decides to donate when faced with an actual choice. Nevertheless, in most cases, the interest in attitudes continues to be closely associated with the ambition of enhancing donation rates [7, 8].

We believe it is time to take a new route in the approach to public attitudes. David Rodríguez-Arias [9] has recently argued for a need to begin focusing studies on the *public acceptability* of strategies used to increase organ donation rates. Noting that only a quarter of the population in Spain supports the country’s presumed consent policy, Rodríguez-Arias suggests that a system should not be measured on its efficacy in procuring organs alone but also on its social acceptability and the transparency of the means by which donations are achieved. To acquire public acceptability, we need to understand what people find important and why [10]. With this paper, we therefore present a study designed to explore public attitudes in order to understand the public acceptability of various options relating to the means by which organ donation is achieved.

With this approach to public attitudes to organ donation, we conducted a telephone-based survey in Denmark among a representative sample of the general population. Denmark is particularly apt for studying acceptability because of its special history with respect to organ donation. Historically, there was prolonged resistance towards the implementation of the brain death criterion in Denmark, and it was adopted only in 1990 after intense public and political debate. In consequence, Denmark is sometimes discussed as an international anomaly with respect to the issue of acceptability of organ transplantation [11, 12]. Interestingly, however, several government studies have indicated dramatic increases in the public support for organ donation [13, 14], and according to the Eurobarometer surveys, Denmark is today one of the European countries with the highest public support for deceased organ and tissue donation [15]. Denmark is therefore an important case for the study of public acceptability. Unlike several other European countries, which have adopted presumed consent legislation, Denmark has maintained the informed consent approach.

## Methods

We developed our questionnaire based on preceding qualitative studies. We conducted 33 qualitative interviews with Danish citizens recruited through online forums for organ donation and through snowball sampling to include individuals with positive, negative and undecided attitudes. The snowball sampling implied asking friends and colleagues to identify people taking a negative attitude towards organ donation, because they proved difficult to recruit (the steps are described in full in [16]). Building on the interviews, we sat as a group and developed survey items covering each of the aspects of our theoretical understanding of attitude. Following Schwarz [17], we understand ‘attitudes’ as hypothetical constructs: attitudes are not taken as simple predictors of behaviour but they operate as key indicators of value-based dispositions which affect how various options are assessed, and they are important in order to understand which policy options best align organ donation and transplantation policies with public acceptability. We have explored attitudes by asking respondents about their overall identification as positive or negative towards organ donation, their personal preferences with respect to the use of one’s own body, the bodies of others, their perceptions of various policy options and their agreement with different forms of reasoning on organ donation. We thereby explored who could take which form of action and still be seen as doing what was deemed acceptable.

Once we had developed a questionnaire to be phoned out to a representative part of the Danish public, we ran two pilot tests (*n* = 10) followed by interviews with the respondents to ensure the validity of each item, and then turned the questionnaire over to the research company Epinion in December 2014. Epinion also implemented pilot testing (*n* = 10) prior to phoning out the actual questionnaire without inserting additional changes. The final questionnaire included a total of 36 questions. It took the phone interviewer approximately 8–10 min to complete it with each respondent. In preparation for the study, we conducted a power calculation on the various potential substrata of the population and decided which variables that would be likely to achieve adequate representation within the sample size we could afford (*n* = 1200). All the data gathered were anonymous when handed over to us and therefore not subject to approval from the ethics committee and data authorities according to Danish law.

The analysis of the data was carried out using IBM SPSS Statistics 22. The answers were given by yes/no answers or on a Likert scale, besides one open-ended question, which was not included in the final analysis. The results are primarily based on descriptive statistics. Responses between groups were compared using the chi-square test, and when testing for the effect of age in scale-based variables, the gamma test was additionally applied. All of our items were correlated with the demographic characteristics presented in Table 1. Background variables are reported in this article only when statistically significant.

**Table 1:** Demographic characteristics of the respondents

	No. (%)
Gender	
Male	591 (49.4 %)
Female	604 (50.6 %)
Age	
18–34	313 (26.3 %)
35–55	434 (36.5 %)
56+	442 (37.2 %)
Education	
Lower secondary (ISCED 2)	366 (30.6 %)
Upper secondary (ISCED 3)	107 (8.9 %)
Post-secondary non-tertiary (ISCED 4)	397 (33.2 %)
Short-cycle tertiary (ISCED 5)	54 (4.5 %)
Bachelor or equivalent (ISCED 6)	184 (15.4 %)
Master or equivalent (ISCED 7)	85 (7.1 %)
Decline to answer	3 (0.3 %)
Religious self-identity	
Not religious	869 (72.7 %)
Christian Protestant	251 (21 %)
Muslim	21 (1.7 %)
Jehovah’s Witness	10 (0.8 %)
Catholic	7 (0.6 %)
Hindu	1 (0.1 %)
Others	9 (0.8 %)
Decline to answer	28 (2.3 %)

## Results

### Population

The study population includes a total of 1195 respondents, see Table 1. A total of 3431 people were called, of which 1402 did not wish to participate and 834 were excluded (reasons: could not be contacted, disease or death, did not speak Danish, or place of residence not part of target group), resulting in a response rate of 46.0 % of the eligible sample or 34.8 % of the total number of respondents called. The respondents were equally allocated in the two genders and had a mean age of 49.4 years (range 18 to 102, SD 18.8). Among those who considered themselves religious (25.8 %; *n* = 308), the most common religious identification was Christian Protestantism.

### Attitudes towards organ donation

Re-using a question asked in 1995, 2001 and 2006 in surveys by The National Board of Health, we asked, ‘What is your general attitude towards organ donation and transplantation?’, and found that 2.2 % stated being negative or very negative, 5.9 % neither negative nor positive and 91.9 % positive or very positive. We correlated this with the age of our respondents and found that the younger age group, aged 18–34 years, was the most positive (95.5 % positive or very positive), followed by respondents aged 35–55 years (93.1 % positive or very positive) and finally the respondents aged 56 years or above (92.1 % positive or very positive).

To gain a deeper understanding of attitudes towards organ donation, we asked our respondents which actions they were willing to take in relation to donating and receiving organs and tissue. Respondents were asked to disregard any potential biological obstacles to donation and consider a manner of death where organ donation is possible. As Table 2 shows, the majority of our respondents stated willingness to take action in organ donation. More than four out of five stated that they would donate their own organs (85 %) and tissue (82.1 %), if they should die under circumstances allowing for donation. The attitudes towards donating an organ or tissue are very similar to the attitudes towards receiving an organ (87.4 %) or tissue (88.6 %).

**Table 2:** Attitude to actions: personal agreement with particular donation actions

	Yes (%)	No (%)	Undecided
Would you be willing to donate your organs after death?	85	7	8
Would you be willing to donate your tissue?	82.1	8.8	9.1
Would you be willing to donate your closest relatives’ organs?	64.7	22.2	13
Would you be willing to donate your closest relatives’ tissue?	66.2	22.5	11.4
Would you be willing to receive organs from a dead donor?	87.4	7.2	5.5
Would you be willing to receive tissue from a dead donor?	86.4	7.6	6
Would you be willing, as a living donor, to donate a kidney to a person of your choice?	85.2	8.2	6.6
Would you be willing to receive a kidney from a living donor?	87.6	7.9	4.5

Fewer, though still a majority, of the respondents, stated that they would also be willing to donate the tissue (66.2 %) and organs (64.7 %) of their relatives. If the respondent answered either ‘no’ or were undecided on the question, ‘Would you be willing to donate your closest relatives’ tissue/organ?’, they were given a sub-question, ‘Would it change your decision if you had knowledge that your relatives wished to donate their organs?’ Of those who answered ‘no’ or were undecided in relation to donating their relatives’ organs or tissue (or both), a significant majority (79 %) stated that it would make them change their answer, thereby allowing for organ and/or tissue donation on behalf of their relatives.

For those willing to donate their own organs and/or tissue, we further asked them if there were any organs or tissues they did not wish to donate. In the Danish organ donor registry, there is an option to register for limited consent and specify any organs one does not wish to donate. Of the respondents who wished to donate (*n* = 1053), the majority (87.1 %) did not have any restrictions to their donation. Of the remaining, our respondents mainly stated reluctance towards donating their corneas (eyes) (5.7 %; *n* = 60), skin (3.4 %, *n* = 36) and heart (2.5 %; *n* = 26).

In Denmark, living kidney donation is possible between relatives, and recently also between friends, and we asked about the respondents’ attitudes towards donating to a ‘person of your own choice’. As with postmortem donation, the majority of the respondents stated willingness to both donate (85.2 %) and receive (87.6 %) a kidney by means of living donation.

### Personal reasoning in organ donation

To gain an understanding of the underlying personal reasoning for the desired action in organ donation, we presented the informants with statements about organ donation encountered in our 33 qualitative interviews, and they were asked to state their levels of agreement. As Table 3 shows, a majority of our respondents (96.6 %) agreed with the statement, ‘I think of organ donation as something you do to help others’. A majority (85.8 %) of our respondents agreed or strongly agree with the statement, ‘I like the idea that my body will be useful after my death’, and if looking only at those also stating a wish to donate their own organs, an even larger majority (92.8 %; *n* = 939; *p* < 0.05) agree or strongly agree with the statement. The majority (83.6 %) of the respondents do not consider organ donation an unpleasant sacrifice. A minority (12.7 %) agree with the statement, ‘I am afraid of not really being dead when the doctors remove the organs’.

**Table 3:** Personal reasoning: attitudes towards value statements about organ donation

	Disagree/strongly disagree (%)	Neither agree nor disagree (%)	Agree/strongly agree (%)	Undecided (%)
I think of organ donation as something you do to help others.	1.2	2.2	96.6	–
I think of organ donation as an unpleasant sacrifice.	83.6	9.7	5.6	1.1
I like the idea that my body will be useful after my death.	5.2	7.3	85.8	1.7
I believe you have the duty to be an organ donor if you are willing to receive an organ.	15.3	14.1	68.6	2
My choice about organ donation reflects what I believe my relatives prefer.	37.4	20.7	35.6	6.3
I am afraid of not really being dead when the doctors remove the organs.	79.3	5.7	12.7	2.3
I find it important that my body goes untouched into the grave.	82.6	7.9	7.9	1.5

In our findings, the majority of the respondents disagree with the statement, ‘I find it important for my body to go untouched into the ground’. However of those (*n* = 84) who do not wish to donate their own organs, a slight majority (51.3 %) agrees with the statement. This indicates that ‘bodily integrity’ might be important for respondents who do not wish to donate their organs. A majority (68.6 %) of the respondents agree with it being a duty to be an organ donor if you are willing to receive an organ yourself.

### Attitudes towards various organ donation policies

In the final section of our questionnaire, we asked the respondents about possible political measures in order to gain an understanding of the political acceptability of certain policies and policy options for regulating organ donation. Since some of the expressions used in the policy debate are not necessarily common public knowledge, we explained the purpose of each policy in the question. The first two questions in Table 4 explore the legitimacy of two different hypothetical consent systems: presumed consent with opt-out and mandatory decision-making. In the survey, the majority (58.6 %) disagree with the statement, ‘Everyone should automatically be considered a potential donor, and those who wish to avoid becoming an organ donor should therefore actively opt-out’, which is how we conceptualized a presumed system with an opt-out option. Even for those stating willingness to donate their own organs or tissue, and for those who stated they were positive or very positive towards organ donation and transplantation, a significant majority (*p* < 0.05) of the respondents disagree or strongly disagree with the statement. The younger-aged group tends to disagree the most with the statement (*γ* = 0.167; *p* < 0.05). We further wondered if those agreeing with the statement, ‘I believe you have the duty to be an organ donor if you are willing to receive an organ’ were also in favour of an opt-out system with presumed consent. However, even among those respondents who agree or strongly agree with having to be an organ donor in order to receive, the majority (52 %; *n* = 425) still disagree or strongly disagree with policies of presumed consent. When asked about mandatory active decision-making, a majority of the respondents agree with the statement that it should be mandatory to register your decision. The younger respondents tend to agree more with the statement (*γ* = −0.190; *p* < 0.05).

**Table 4:** Political legitimacy: attitudes towards policies regulating organ donation

	Disagree/strongly disagree (%)	Neither agree nor disagree (%)	Agree/strongly agree (%)	Undecided (%)
Everyone should automatically be considered a potential donor, and those who wish to avoid becoming an organ donor should therefore actively opt out.	58.6	8.5	30.4	2.5
It should be mandatory by law for everyone over the age of 18 to decide whether they want to be an organ donor, and to register their decision in the organ donor registry.	24.6	9.2	63.9	2.3
It should be possible to motivate donors or relatives of potential donors with money, to make them donate organs.	87.9	5.4	5.8	0.9
It would be fair if donors or relatives received compensation for any potential expenses in relation to the donation.	32.9	11	52.7	3.4
The health services must mediate the contact between the relatives of a deceased donor and the recipient of the organ, if both parties request it.	16.9	18.5	60.3	4.3
There is too much being done to promote organ donation already.	87.4	7.5	2.3	2.8

Another policy option considered to promote organ donation is the use of monetary incentives. To explore the acceptability of initiatives in organ donation, we included two questions focusing on money as motivation versus money as compensation. Very few (5.8 %) found it acceptable to use money as a motivation for donating organs, while a slight majority (52.7 %) agreed with the statement, ‘It would be fair if donors or relatives received compensation for any potential expenses in relation to the donation’. In both of these questions on financial initiatives, women tended to disagree more with the statements than men (*p* < 0.05).

Another policy issue, which has received substantial attention, relates to rules regulating contact between donor relatives and recipient. In contrast to current policies upholding strict anonymity, a majority of our respondents (60.3 %) found it acceptable to make it compulsory for the hospital to mediate this contact, if both parties want it.

Finally, we asked if there is already too much being done to promote organ donation, partly in an attempt to accommodate individuals who did not feel their attitude had been clearly expressed through previous questions and partly to triangulate and capture preferences for fewer rather than additional initiatives. A minority of 2.3 % of our respondents agreed with the statement.

### Religion

We were interested in whether religious self-identity influences attitudes towards organ donation. As Table 5 shows, the majority of the respondents are positive towards organ donation, regardless of whether they consider themselves religious or not.

**Table 5:** Religious attitudes: influence of religious self-identification on attitudes towards organ donation

Question	Answer	Religious (%)	Not religious (%)
What is your general attitude towards organ donation and transplantation?	Very positive/positive	89.6	93.2
	Neither negative nor positive	6.8	5.1
	Very negative/negative	3.6	1.6
	Total	100	100
Would you be willing to donate your organs after death?	Yes	76.7	88.1
	No	11	5.6
	Undecided	12.3	6.2
	Total	100	100
Would you be willing to receive organs from a dead donor?	Yes	76.7	91.4
	No	11.4	5.8
	Undecided	11.7	2.9
	Total	100	100

## Discussion

### Main findings: overall attitudes

**Fig. 1:**
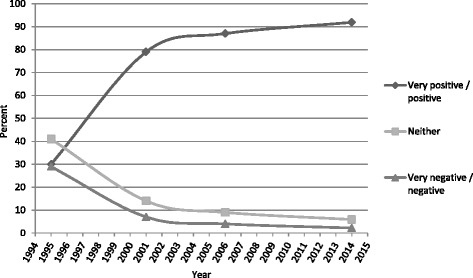
Historical development in the general attitude towards organ donation and transplantation based on this and previous surveys using the same question in 1995, 2001, 2006 and this study. Percentages add up to 100

We re-used a question asked by the Danish Health and Medicine Authorities in 1995, 2001 and 2006 [13, 14] in our 2014 questionnaire, and Fig. 1 draws upon both our own and the historical data to demonstrate the historical developments in public attitudes. The figure shows a trend of increasingly positive attitudes towards organ donation and transplantation and also that the population is increasingly clarified in its stance towards this medical technology.

The question we could use to identify historical developments was complemented by other, less ambiguous questions covering different aspects of an attitude. They further document positive attitudes towards donating as well as receiving organs. People were more positive towards donating their own rather than the organs of relatives, as has also been found elsewhere [18]. Though religion has been presented as a barrier to donation in interviews with Danish healthcare professionals as well as in international surveys, e.g. [4], religious self-identification does not significantly influence attitudes towards donation in a negative sense in our survey.

### How should the findings be interpreted?

When considering our results, a pattern seems to emerge, a pattern in which self-determination is central for public acceptability, as discussed in the following. We have already noted that people are more positive towards donating their own organs than the organs of their relatives. Furthermore, they will change their negative stance if the positive donation wishes of their relatives are known so that their autonomy is respected. Also, there is limited support for a presumed consent system with opt-out and much higher support for a system demanding mandatory registration of personal decisions. Studies in other European settings have found a greater support for an opt-out model [19, 20]. It is especially noteworthy in our results that even those who are positive towards organ donation are negative towards presumed consent and also that the majority of those who agree with the statement that one ought to be willing to donate in order to receive still disagree with a presumed consent system. Moreover, the younger, who are most strongly in favour of organ donation, are also strongly opposed to a presumed consent system.

Monetary incentives are associated with even more limited acceptability than presumed consent as a way to promote organ donation, though with a clear difference between attitudes to money used as incentives and as compensation (see also [21]), as well as the Eurobarometer [15]). Finally, we found that a majority agrees or strongly agrees with the statement that the healthcare system should help facilitate contact between organ recipient and donor family, if both parties wish for it, despite the current policy of strict anonymization. Again, it indicates that the public is more positive towards policies that facilitate choice than towards policies that promote particular values, irrespective of agreement with the actual values themselves.

The overall pattern that comes across is thus that the high level of public acceptability and the positive attitudes prevailing in Denmark depend on a system allowing active, individual decision-making. With this understanding, we might also consider a new explanation of what could appear as a paradox: when asked in surveys, Danes are very supportive of organ donation; but Denmark has relatively low deceased donation rates of 13.8 donor per million in 2014 compared to its Scandinavian neighbours: Sweden, Norway and Finland with donation rates of 17.1, 22.6 and 21.9, respectively, in 2014 [22]. However, the low Danish donation rates primarily reflect a high family refusal rate (20 % in 2012, 42 % in 2013 and 32 % in 2014) [23–25]. Hence, there might be no major gap between attitude and behaviour [5, 6]: respondents want to donate their own organs but not the organs of their relatives, unless they know this is what the deceased would have wanted. It could imply that to promote donation rates in a publically acceptable way, the way forward would be to ensure that people record a choice.

If we wish to ensure the long-term social sustainability of organ donation and transplantation, it will be important to think not only about what might increase donation rates but also about what makes donations meaningful to donors. Therefore, it will be essential for policymakers in Denmark and elsewhere to monitor public attitudes and take issues of acceptability into account when considering new initiatives to promote organ donation. To find out whether the strong emphasis on individual decision-making is a particular Danish trait, it would be useful to explore this further in other national settings in future studies of the public acceptability of organ donation.

### Representativity

We acknowledge that survey instruments involve weaknesses when measuring hypothetical choices and emotionally complex issues. We therefore began our study with thorough qualitative work. Furthermore, we abstain from any attempt at predicting donor behaviour. We use attitudes to establish an indication of acceptability. The validity of the questions was tested in the qualitative work and the pilot tests. The telephone-based methodology makes it difficult to properly assess dropout rates because we cannot know why people do not pick up their phone. However, with respect to gender, age and educational level, the respondents were representative for the general Danish population. Also, the preferences given for the selected organs (cornea, skin and hearts) are similar to those recorded in the Danish organ donor registry. The overall number of people identifying themselves as religious is lower than the average membership of religious denominations [26], but previous studies indicate that only a minority of the Danish population consider themselves religious even when they are members of a denomination [27]. From that perspective, our sample is therefore likely to be representative of the general adult population.

## Conclusions

Whereas multiple studies have explored attitudes in order to enhance donor motivation for organ donation, we have explored attitudes with the aim of understanding public acceptability because it is central for the long-term sustainability of this medical technology that the policies regulating it are aligned with the dominant values and priorities of the public. We have demonstrated a remarkable growth in the public support for organ donation in Denmark and identified a pattern in the values that sustain this support, namely self-determinacy. As such, policies fostering individual choice are met with positive attitudes, while presumed consent and monetary incentives are met with negative attitudes. Our approach calls for comparative studies in other countries to generate a better overall understanding of the conditions of acceptability, which need to be in place to ensure the long-term social robustness of organ donation and thereby safeguard this important medical technology.
